# Root Response to Drought Stress in Rice (*Oryza sativa* L.)

**DOI:** 10.3390/ijms21041513

**Published:** 2020-02-22

**Authors:** Yoonha Kim, Yong Suk Chung, Eungyeong Lee, Pooja Tripathi, Seong Heo, Kyung-Hwan Kim

**Affiliations:** 1 School of Applied Biosciences, Kyungpook National University, Daegu 41566, Korea; kyh1229@knu.ac.kr (Y.K.); pooja@knu.ac.kr (P.T.); 2 Faculty of Bioscience and Industry, College of Applied Life Science, SARI, Jeju National University, Jeju 63243, Korea; yschung@jejunu.ac.kr; 3 National Institute of Agricultural Sciences, Rural Development Administration (RDA), Jeonju 54874, Korea; wowlek44@korea.kr; 4 Ganghwa Agricultural Technology Service Center, Incheon 23038, Korea; sycarus@korea.kr

**Keywords:** root morphological trait, root architecture, physiological response to drought, screening methods for drought stress, phenomics

## Abstract

The current unpredictable climate changes are causing frequent and severe droughts. Such circumstances emphasize the need to understand the response of plants to drought stress, especially in rice, one of the most important grain crops. Knowledge of the drought stress response components is especially important in plant roots, the major organ for the absorption of water and nutrients from the soil. Thus, this article reviews the root response to drought stress in rice. It is presented to provide readers with information of use for their own research and breeding program for tolerance to drought stress in rice.

## 1. Introduction

Rice (*Oryza sativa* L.) is grown in a wide range of ecosystems, including flood- and drought-prone environments [1]. As rice is the main food for more than half of the world’s population, rice yield losses pose a major threat to food security [1]. Rice is vulnerable to a wide range of abiotic stresses, like drought, heavy metals, salinity, cold, and submergence [2]. It is a high water-consuming crop and irrigated rice represents 53% of the global cultivated area of rice [3]. Availability and accessibility of fresh water determine the global rice production [4].

During the various stages of growth and development, plants are persistently likely to face various abiotic and biotic stresses. Management practices can have a notable impact on plant responses to biotic stress, whereas abiotic stresses, like extreme temperature, UV, and excess or deficient water in soil is a dominant factor limiting crop productivity under field conditions [5,6,7,8]. The plant organs, such as leaf and root, orchestrate defense mechanisms (internal or external) in response to abiotic stress [9,10,11]. Root is the first organ exposed to water stress because water stress results from an insufficient or excessive level of water in the soil [7,8,12,13,14].

In this review, we describe the genetic, proteomic, and morphological responses of root to drought stress in rice. Additionally, we introduce a root phenotyping method as a phenomics tool for evaluating drought tolerance in field experiments.

## 2. Global Status of Drought Stress

Drought is a natural phenomenon caused by the combinations of hydrological, climatic, and environmental forces that result in insufficient precipitation for agricultural production over a prolonged duration [15]. Drought severity is of immense concern because of its extensive impacts on the world [16]. The frequency, severity, and long-term trends of global drought remain contentious [17,18], yet the incidence and extremity of drought have been increasing globally, such as in the Mediterranean [19], Central China [20], and West Africa [21]. Drought is a major constraint to food production worldwide, as it can occur for varying lengths of time and intensity at any stage of crop growth and development [22]. With the increasing human population and depleting water resources, the development of drought-resistant crops is of prime importance to preventing crop yield losses from drought stress [22]. In plants, roots are the key organ for absorbing water and nutrients.

## 3. Drought Stress

Rice breeding programs have largely focused on understanding the plant’s response to various abiotic and biotic stresses to enhance yield [23]. The major constraint of rain-fed rice production is drought [24]. Breeding for high-input irrigated conditions favors shallow root systems to acquire the resources from the top layer of the soil, whereas breeding programs for low-input rain-fed conditions tend toward a deep and robust root system, needed to extract the water and nutrients from a large volume of soil [1]. Three common types of drought affect rice production: early water stress that causes a delay in seedling transplantation, mild sporadic stress having cumulative effects, and late stress affecting late-maturing varieties [25]. Drought stress induces various physiological and biochemical changes in rice at different developmental stages [26]. It is thought that the ability of the plant to modify its roots to grow thicker and deeper into the soil might be an important mechanism to avoid drought stress, and there is ample evidence that assimilates are relocated to roots instead of shoots as a response to water stress [27]. On the contrary, some research demonstrated that root growth in rice decreases under drought stress [28]. These findings show that the response of roots to water stress is highly dependent on the crop genotype, and period and intensity of stress [29]. The impact of drought stress on rice yield also depends on the growth stages, with mid-tillering, flowering, and panicle initiation identified as the most sensitive stages [30].

### 3.1. Root Function for Water Uptake

Roots acquire water and nutrients from the soil. Hence, the morphological and physiological characteristics of roots play a major role in determining shoot growth and overall production [31]. The access of water to a plant is determined by its root system, properties, structure, and distribution, thus improving root traits to increase the uptake of soil moisture and maintain productivity under water stress is of huge interest [32,33]. Herbaceous plants have a root system comprised of coarse roots, which include the primary roots that originate from the tap root system and the nodal/seminal roots of fibrous root systems, easily distinguishable from the finer lateral roots [34]. Coarse roots provide anchorage to plants and determine the root depth, architecture, and depth of penetration into the soil layers [35]. Changes in the metrics of root-to-shoot relationships can compensate for moisture deficiency and maintain stomatal conductance under drought stress conditions [36]. The optimal partitioning theory proposes that a plant distributes the resources among its various organs for optimal growth [37]. It further suggests that the shoot ratio and some degree of responsiveness may change the ratio to balance the resources that limit plant growth even though the plants are adapted to produce a certain root [38]. Roots with a small diameter and a high specific root length increase the surface area of roots in contact with moisture, increasing the soil volume that can be explored for water, and also increase the hydraulic conductance by decreasing the apoplastic barrier of water entering the xylem [39,40]. In addition, the decrease in root diameter also helps to enhance water access and increases the productivity of plants under water stress [41].

Other root morphological characters influencing resource acquisition are an increase in the number of fine roots and the rates of overall root growth [42]. Root hairs increase the contact area of roots with soil particles and thereby aid in absorbing soil water [41]. Root hairs in many plants are associated with improved accumulation of water and nutrients, as well as responsiveness to stresses [43]. By contrast, root hairs were found to be vital for nutrient uptake but had no notable role in water absorption in rice [44,45]. Rice roots under water stress are affected by aquaporin expression [46], and this is directly associated with root hydraulic conductivity [47]. Aquaporins regulate the water transport capacity of the root system (i.e., the root hydraulic conductivity) to meet the water demand of plants [48,49]. Extreme water deficits limit root growth and development because of the increased soil resistance and low water availability [50]. The consequent decrease in root surface area is compensated for by the production of root hairs and aquaporins [48,49].

The continuous growth of new root tips may be vital for the uptake of water and nutrients [51]. Although the root length and surface area may determine the uptake of soil resources [52], young root tips are the main regions of water uptake [53]. The diameter of xylem vessels also influences the root hydraulic conductivity and ultimately determines the plant productivity under drought stress [54]. Plants with a low xylem diameter generally have lower hydraulic conductivity and a lower risk of cavitation from more conservative water use relative to those with a higher xylem vessel diameter [55], with some exceptions [56]. Breeding strategies to reduce the root xylem diameter will cause a reduction in hydraulic conductance under sufficient moisture availability. Consequently, breeding programs have focused their research on roots that absorb water, especially under drought conditions [57]. Exceptional plant species that are capable of maintaining high rates of transpiration and conductivity, and that exhibit high resistance to cavitation have been found [56]. Understanding such mechanisms may be of importance to breeding programs to gain maximum yield potential during suitable growing conditions [33].

### 3.2. Root System Architecture under Drought Stress

Roots are the primary plant organs to detect soil condition alterations, with a vital role in response to water stress [31]. There is ample evidence that the yield of cereal crops grown under water and nutrient deficiencies can be increased by altering the root structure because this improves their ability to capture soil resources [58]. Measurement of root systems in rice under drought stress revealed a positive correlation of root diameter and depth with plant vigor [27]. Drought avoidance in many upland *japonica* varieties of rice is accomplished by extensive and deep root systems, whereas *indica* subspecies typically shorten their growth period [59].

Root architecture is known to be a primary aspect of the root system to acquire soil resources. Rice roots vary genetically in thickness and penetration ability [60]. In comparison to other cereal crops, rice has poor adaptation to water-scarce conditions. Rice absorbs very little or no soil water at 60 cm depth [61]. The deep rooting system in upland rice varieties is considered effective in maintaining yield under drought [61]. Rain-fed lowland rice faces fluctuating soil water conditions, and some rice genotypes exhibit adaptation to such conditions by increasing root growth before and during the early stages of drought [62]. Rain-fed rice can also penetrate hardpan, an ability critical for establishing a deep root system to improve adaptation to drought stress [63].

Root structure, function, and movement depend on the soil moisture content, and the root density in the subsurface horizon determines the root response to drought stress [64]. For short-statured plants, like rice, wheat, beans, and plants that grow in limited water conditions, a deep root system for acquiring moisture from soil profiles is found to be beneficial [35]. Root length densities from 0.5 to 1 cm^−3^ are usually adequate to meet moisture demand in plants [65], but a higher root surface area density is needed to prevail over hydraulic resistance in dry soil. To increase the surface area for water uptake, soil with low moisture induces increased allocation of the assimilates to the roots [66] and modifies carbon assimilation in the roots, which leads to increased growth toward the water-sufficient soil layers [67]. In addition, root hairs enhance the root surface area for water uptake by absorbing water from fine pores that are inaccessible to root apex [68]. The number of dividing cells in root tips and the characteristics of root caps have been associated with root survival during water stress [69].

The efficiency of water uptake from a heterogeneous soil setting depends on the root architecture. Therefore, an improved understanding of the response of roots to different levels of moisture stress is a vital aspect of plant biology [70]. When the soil has dried, roots cannot uptake or sometimes even cause water loss to the soil, leading to reductions in osmotic potential and matric potential [53], with consequent decreases in turgor pressure and cell volume [71]. To overcome severe water shortage, the root cells must activate mechanisms to tackle water loss and its related effects. Sometimes, the cell solute potential is decreased, which increases turgor pressure and sustains growth in water-deficit environments [70].

Severe water stress in rice accounts for economic yield losses of 48–94% in the reproductive stage [2] and 60% in the grain-filling stage [72]. Drying of the soil surface layer might lead the roots to seek moisture available deep in the soil profile. Breeding for plants with less root length density in shallow layers of soil, and high root length density in medium- and deep-layers, has been considered an efficient water management strategy [41,73]. The hierarchical structure of the root system may promote hydraulic lift, facilitating water uptake from deep soil profiles [74]. When deep root systems can increase crop productivity, large-diameter xylem vessels may be beneficial to increase the axial hydraulic conductivity of roots growing in deep soil layers [41]. With the advancement in breeding strategies to develop drought-tolerant crops, focusing on the whole plant and root characteristics, and studying patterns of root growth in varying locations and time is advantageous [33].

## 4. Physiological Responses of Roots to Drought Stress

Three adaptive mechanisms have been confirmed for rice under drought stress: (i) osmotic adjustment in roots, if possible, in conditions with a relatively small soil water reservoir, (ii) increased root penetration into the soil, and (iii) increased root density, depth, and the root-to-shoot ratio in conditions with a relatively large soil water reservoir [63]. It has been claimed that rice plants with deep roots are more tolerant to water stress and maintain productivity in such circumstances [75]. When water deficiency occurs, root growth is favored over shoot growth. When water potential is reduced, osmotic adjustments in the root system aid in maintaining some level of turgidity, and the water potential gradient is re-established for water uptake. These adjustments are responsible for the growth of roots under low water potential [76]. Stomatal closure is another mechanism that plants use to cope with water stress. This action reduces leaf moisture loss and decreases the gaseous exchange between the plant and the atmosphere, which impacts the rate of photosynthesis and ultimately reduces the yield of the crop but allows survival of plants under drought stress in the short term [77]. The regulation of stomatal conductance is not properly understood, but it could be a result of low root moisture status that is communicated to the leaf by hormone signaling [78].

The bleeding rate of sap from the root system was substantially different between drought-tolerant and drought-susceptible rice genotypes [79]. Lateral root formation increased under water stress, which increased the surface area for water absorption from shrinking water columns. There was also a marked reduction in the nodal root diameter, leading to relatively finer roots to conserve resources. The increased root cross-sectional diameter, represented by the stele diameter, was considered for prioritizing water retention in vascular tissue instead of reducing radial oxygen loss as drought ensues [79]. The decreased risk of xylem vessel cavitation under drought stress was attributed to the decreased diameter or number of xylem vessels [79]. The sclerenchyma cell diameter increased under drought stress because closely packed cells are not required for oxygen retention at the time of drought [79]. Aerenchyma cell formation decreased under drought stress, as these cells are required mostly for supplying oxygen in flooded soils [79]. Daytime changes in hydraulic conductivity and sap bleeding rate were observed in rice roots, with all genotypes exhibiting reduced levels at night, and varying levels in the early morning and mid-day [79]. Drought-resistant genotypes develop these traits to facilitate water uptake at times of the day when transpiration is most efficient [79]. Finally, differential trends in the synchronization of diurnal changes in root hydraulic conductivity and leaf water potential between genotypes were identified, which might increase the water use efficiency in plants [79].

### 4.1. Phytohormones on Stress

The expression of ethylene response factor JERF1 improves drought resistance in rice [80]. Overexpression of JERF1 improves drought tolerance of rice seedlings, increases the proline content of rice, decreases water loss in transgenic rice, activates the expression of genes that respond to stress, increases abscisic acid (ABA) synthesis, and regulates the expression of ABA synthesis genes in rice [80]. Various transcription factors, like MYB, basic helix-loop-helix, ethylene response factor, basic-domain leucine zipper, and homeodomain, are involved in the regulation of ABA-dependent signaling pathways [81,82] and exert a major role in the stress response by regulating the expression of many downstream drought-responsive genes. In addition, other hormones, particularly cytokinin, salicylic acid, and jasmonic acid, directly or indirectly affect the abiotic stress response [83]. Under drought stress, cytokinin levels decrease. Furthermore, various genes that encode proteins linked with the cytokinin signaling pathway were affected differently by abiotic stresses [84]. Exogenous application of jasmonic acid to crops under drought stress increases the activity of antioxidant enzymes [85]. Likewise, auxin is necessary for root development, and any disturbance in its synthesis, signaling, or transport affects the development of the root system [86]. For instance, a mutation in the putative auxin influx carrier gene *OsAUX1* led to a decreased number of lateral roots, while its overexpression had the opposite effect [87]. Certain modifications in the synthesis, transport, and signaling of auxin profoundly affect drought resistance in rice. For example, overexpression of auxin efflux carrier gene *OsPIN3t* [88] or *OsGH3.2* [89] and *OsGH3.13* [90] or the *auxin/IAA* gene *OsIAA6* [91] that targets auxin receptor TIT1, resulted in improved drought tolerance. Overexpression of *YUC* genes in rice leads to the production of several adventitious roots [92]. It suggests that the induction of the *YUC* gene in rice under drought stress results in auxin production and, subsequently, increases the number of roots, helping rice plants to adapt to harsh environments.

### 4.2. Osmoregulation

Cells remain turgid by inducing the accumulation of solutes and reducing the osmotic potential [93]. Osmoregulatory substances can be either inorganic ions, such as K^+^ and Na^+^, or organic matter that adjusts the cytoplasmic osmotic potential, like betaine and proline, for example [94]. Since the discovery of proline accumulation in 1954, its relation to drought stress has been studied extensively [95]. Proline is a non-protein amino acid that acts as an osmoprotectant, serves as an energy sink to regulate the redox potential [96], and reduces cell acidity [97]. Proline metabolism was found to be strongly responsive to certain carbohydrates, especially when the intercellular concentrations exceeded a certain threshold, thought to occur as a result of water-deficit stress [97].

## 5. Genetic Mechanism of Drought Stress

Plants often face adverse environmental conditions. To cope with these environmental stresses, plants execute various physiological and metabolic responses, such as the expression of stress-responsive genes and the synthesis of functional proteins [98]. Drought tolerance involves responses at the whole plant level, allowing the integration of shoot growth, root architecture, and the transpiration, water absorption, and growth rate responses [99]. Several genes and quantitative resistance loci associated with root response to various stresses were identified. *PUP1*, a root quantitative trait loci (QTL) associated with phosphorus uptake [100], and *DEEPER ROOTING 1* (*DRO1*), a QTL associated with root depth, have been cloned [75]. The accuracy of the number and location of the identified QTL have been refined by recent developments in meta-QTL analysis and genome-wide association mapping approaches [101]. Nonetheless, strategies for drought tolerance in rice have attained limited success because of knowledge gaps in root growth at the molecular level, partially because root phenotyping is laborious and time-consuming [102].

### 5.1. Genetics of Root Traits under Drought Stress

Multiple genes with small effects control for most of the root traits and the effect of their interaction varies with the prevailing environment [103,104]. Root traits can be challenging to identify, leading to a preponderance of genetic research aboveground compared with that belowground [105]. The need for rice plants to be adapted to various water conditions has encouraged the identification of genes and QTL that determine root structure, development, and functioning [1]. The development of experimental setups that imitate actual field conditions has helped to develop diverse phenotyping platforms for screening important root traits, mutant resources, and mapping populations [1]. A search of the database TropGene for QTL associated with drought stress in rice revealed 139 QTL in just five studies for root traits, whereas 387 QTL were identified for non-root traits in 15 studies [106]. As phenotyping for genetic research is usually accomplished in controlled environmental conditions, cautious interpretation of such processes is required in root studies, as a lack of quantitative or qualitative phenotypic information may lead to inconsistencies in QTL and gene locations [107,108,109]. The genetic variation in the capacity of rice to grow deep roots has been linked with its productivity under water-stress conditions. For example, the rice *DRO1* gene on chromosome 9, functions downstream of auxin signaling and increases the growth of root tips in response to gravity [75]. Introgression of this gene by backcrossing to rice variety IR64 showed increased drought resistance without yield reduction in well-watered conditions. QTL that regulate root system size and plasticity have been identified in the model plant *Arabidopsis thaliana* [110,111]. Enhanced water uptake linked with deep root architecture and a specific root length was associated with a QTL in rice that also provides yield improvements under extreme water deficit [112]. Mapping QTL of root traits strongly connected with drought-resistance mechanisms provides potential strategic information for marker-assisted selection (MAS). Much information on QTL and markers is confounded by insufficient phenotyping and provides erratic contributions covering various populations and environments or makes only a small contribution to the important trait [107]. QTL discovered in controlled environmental conditions must be tested in field conditions and should contribute to crop improvement before being used in MAS programs, which explains why few reports have been used for root characteristics in plant breeding programs [33]. The highly drought-tolerant upland rice cultivar Birsa Vikas Dhan 111, which was selected for larger root architecture, was successfully developed by marker-assisted backcrossing breeding targeting five donor-parent chromosomal regions, one associated with end-use quality and four related to root traits [113]. For this, numerous markers were selected for maintaining the recurrent parent background [113]. As the variation in root structure is difficult to phenotype, MAS provides the best options of combinations of above- and belowground traits [33]. The use of molecular markers that are important in drought tolerance has been challenging to researchers [33]. However, molecular markers showing strong linkage disequilibrium with desired QTL for root traits or genes must be identified for MAS to be successful in breeding programs [33].

A recent development in root phenotyping of *japonica* rice is a non-destructive process involving X-ray computed tomography [114], which captures the entire root system in soil pots in situ using transparent media that mimics field conditions [115]. This hydroponic-based system includes a rhizoscope made of plexiglass sandwiches filled with glass beads, imitating soil resistance. Likewise, image analysis of histological sections of root could provide valuable information on the capability of rice to survive water deficit by examining radial tissue differentiation [79]. Deciphering the functions of rice genes, particularly those with a key role in agronomic traits, will require sharing rice mutant resources because multiple genes associated with roots differ in their function and expression according to location and time [116].

### 5.2. Genetic Mechanisms Governing Drought Tolerance

The extent of drought tolerance capacity of a plant depends on the presence and efficiency of drought-adaptation mechanisms within its genome [117]. With the domestication of crops and reduction in genetic diversity, deleterious mutations may have been promoted in stress response mechanisms of crops [117]. When plants face drought, the concomitant cellular dehydration causes a reduction in cytosolic and vacuolar volumes, inducing osmotic stress [118].

Overexpression of *EcNAC67* increases drought resistance in rice. Under water stress, transgenic plants exhibited a delay in leaf rolling symptoms, revived rapidly upon re-watering, and maintained approximately 20% higher relative water content in the leaves and lesser decrease in plant height and yield when compared with non-transgenic ASD16 plants [119]. No phenotypic abnormalities were observed in the transgenic plants, indicating *EcNAC67* as a source for developing crop resistance to drought stress [119].

In rice, *DSM1* is a Raf-like MAPKKK gene that encodes a putative mitogen-activated protein kinase kinase. *DSMI* mutants displayed a drought-responsive hypersensitive phenotype, suggesting that *DSMI* might mediate drought responses in rice by regulating the scavenging of radical oxygen species [120]. In other work, transgenic ASD16 rice plants (shallow-rooted) overexpressing *OsARD4* exhibited the drought-adaptive traits of the rice genotype Nootripathu (deep-rooted), including its high root bulk [121]. Growth maintenance of roots triggered by drought stress is an adaptive strategy for water uptake. In response to water deficiency, expansin genes that are associated with cell expansion and cell wall loosening were shown to alter their expression pattern [122,123].

### 5.3. Molecular Level Responses to Drought Stress

Drought stress in rice, like many plant species, activates the ABA-dependent signaling pathway [124]. It has been documented that OsPYL/RCAR5 is a functional cytosolic ABA receptor positively regulating abiotic stress-responsive gene expression, and overexpression of the *OsPYL/RCAR5* gene has improved drought tolerance in transgenic rice [125]. Experiments have shown that rice DREB transcription factors also work as vital regulators in ABA-independent drought responses [126]. Out of five *DREB-2* type genes, *OsDREB2A* and *OsDREB2B* are upregulated by abiotic stress. *OsDREB2B* generates *OsDREB2B1* and *OsDREB2B2* transcripts [126]. High or low temperature, drought, and salinity stress caused accumulation of *OsDREB2B2* transcripts, whereas *OsDREB2B1* transcripts varied only for cold stress, indicating that *OsDREB2B2* has a key role in the abiotic stress response in rice through the alternative splicing system [126]. Unlike other *DREB-1* type genes in rice, *OsDREB1F* regulates the ABA-dependent pathway, and rice plants that overexpress this gene have increased drought tolerance [126]. Table 1 provides a summary of the genes involved in drought resistance in rice.

Other than those mentioned above, more than 5000 genes are upregulated, and over 6000 genes are downregulated by water stress in rice [127]. High expression levels of genes encoding malate synthase and isocitrate lyase in the glyoxylate cycle occurred, along with increased glucose levels, in rice under various abiotic stresses [127]. The decreased cytokinin level was correlated with reduced expression of the cytochrome P450 *735A* gene [127]. On studying the expression profiles of drought-responsive genes in both drought-susceptible and drought-tolerant rice genotypes, it was observed that senescence-related degradation and photosynthesis-related gene expression were decreased in drought-tolerant cultivars compared with those in drought-sensitive cultivars [129]. Through integrated analyses of gene expression and stress tolerance, marker transcripts for selection of drought tolerance have been identified in a wide range of rice germplasm resources using the comprehensive expression data [129]. The expression level of the marker transcripts under drought stress was correlated with drought tolerance [129]. In another study, 5284 drought stress-responsive genes were identified [130]. A comparison of the drought-responsive genes in *indica* rice genotypes having contrasting drought tolerances revealed an upregulation of the α-linoleic acid metabolic pathway in drought-tolerant genotypes [131]. Results of the genome-wide distribution pattern of histone H3 lysine 4 tri-methylation demonstrated a positive correlation between the levels of methylation and the expression levels of some of the drought-responsive genes [132]. Several other studies have equivocally demonstrated the relevance of root traits with water uptake. When five segments on different chromosomes were introgressed into rice lines, including four segments carrying QTL for improved root length and thickness, and one carrying a recessive gene for aroma, only the target segment on chromosome 9 caused a marked increase in root length under irrigated and drought stress conditions [113]. Different combinations of the QTL contributed positively in different test environments and promoted water uptake, highlighting their importance in crop improvement programs for drought tolerance in rice [103,133]. Overexpression of *OsNAC5* was used to develop a transgenic rice line with increased root length, which increased the yield by 9–26% [128]. A field trial of upland rice introgressed with root QTL resulted in plants with increased root length and a yield benefit of 1 t/ha in comparison to the control [113]. Large data sets of rice, however, indicate there are limited studies on signaling cascades associated with drought stress responses.

Many studies are focusing on the molecular control of lateral root branching in various mutant rice lines [134], and the signaling pathways and genes that control the drought response in rice [135]. The screening of various rice germplasms for root characteristics linked with drought tolerance has proven beneficial in several breeding projects [35]. For studying root traits associated with drought, selected germplasms comprise those with relatively thicker root systems, thicker root systems, early maturity, and the capacity to produce new tillers after re-wetting [136].

## 6. Effects of Drought on Plant–Soil Microbe Interactions

The soil microbial population influences plant assembly, biodiversity, and the ecosystem [137]. In the coming century, we can expect increasing temperatures and changes in the global climate pattern. These phenomena might change the soil microbial distribution and the outcome of plant–microbial interactions. Therefore, understanding the dynamics of soil microbes on various types of abiotic stresses may be beneficial to prepare for the near future [138]. Spatial variation in abiotic factors and the biotic environment largely determine the soil microbial distribution [139]. Plants adapt to abiotic [140] and biotic soil conditions [141], and soil microbes adjust to the plant genotypes [141]. Likewise, any variation in the microbial species composition and distribution affects plants positively or negatively [142,143]. The outcome of species interaction is altered by climate change effects [144]. For example, fungal communities have been altered because of changes in soil moisture and temperature [145]. It has been reported that drought stress increases root colonization by arbuscular mycorrhizal fungi [146], representing an important strategy for water stress management [147]. Some research concluded that a change in precipitation does not always affect the arbuscular mycorrhizal fungal colonization [148], but the community composition is affected [148,149]. Starvation, osmotic stress, and competition for resources create pressure on the distribution and functioning of the soil bacterial population [150]. Soil microbes secrete various enzymes important for soil nutrient cycles and fertility [151]. Decreases in microbial biomass and ATP values caused serious damage to the microbial community under drought as compared with sufficiently irrigated soils [152]. Furthermore, a pulse of CO_2_ occurs when dry soil is watered by rainfall [153], which contributes to heterotrophic respiration in the ecosystems [154]. Further research is required to understand how changes in soil carbon cycling during drying and wetting of soil affect microbial respiration [155]. Several studies investigated the impact of drought stress on the composition, population, and functioning of soil microorganisms and concluded that drought severity depletes the activity of soil microorganisms [156,157]. In addition to this, the carbon-to-nitrogen ratio of the soil also decreases with drought severity [158].

## 7. Screening Methods for Identifying Root Traits Associated with Drought Stress

Among the measurements of root dry mass, root length is often used as a direct evaluation and can predict rice yield [159]. Root pulling resistance is also considered to be linked with biomass, as well as root thickness and branching, and is an indirect approach to screen drought-tolerant genotypes with large root systems [160]. Root xylem vessels were also used to screen drought-tolerant genotypes [161]. The distribution of xylem vessels is said to be dramatically different between upland and lowland cultivars, as upland rice is claimed to possess more root xylem vessels at the tip and mid-section of the roots as compared with upland rice [162]. Rice root characterization is often performed in containers in greenhouses and fields, which is labor- and time-intensive. To overcome these issues, various root imaging techniques have been developed to investigate the dynamics of root systems and provide opportunities that improve the precision of studying the genetics of root traits. Non-invasive imaging analysis discloses information on spatial root distribution and helps to elucidate the genetic control of rice root architecture [33], as summarized in the following section.

### 7.1. Process of Two-Dimensional (2D) Imaging Phenotyping

#### 7.1.1. Selection of Imaging Platform

**Figure 1:**
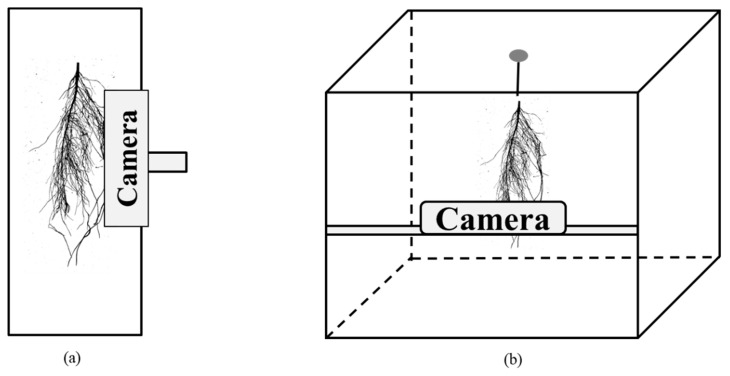
Two methods of collecting two-dimensional images. (**a**) horizontal root crown method in which the roots are placed on a flat surface, and the camera is placed above the roots; (**b**) vertical root crown method in which the root is hung from the roof and the camera is placed in front of the hanging root.

To collect 2D images, a platform must be selected. The two different methods of collecting 2D images are termed horizontal and vertical root crown methods [163]. These methods differ in the position of the object. In the horizontal root crown method, root samples are placed on a flat surface, then an image is captured by a camera placed parallel to the flat surface (Figure 1a). In the vertical root crown approach, the roots are connected to the roof of a rhizobox, and then images are collected by a horizontally-attached camera (Figure 1b).

#### 7.1.2. Digging the Root Samples, Image Collection, and Analysis

The root architecture in rice governs the crop performance under drought [164]. Hence, deep rooting is one aim of many rice improvement programs [165]. Varieties with comparatively greater root length density in deeper soil layers and thicker coarse roots are preferred among upland varieties [113], whereas thick, coarse roots to penetrate hard soil layers are preferred in lowland varieties [79]. The growth of lateral or fine roots has also been reported to enhance water absorption and maintain productivity under water-deficient conditions [150]. Thus, it is important to screen the root morphology.

For image-based phenotyping of root crowns, a mark is made around the target sample, and then the root crown is carefully removed from the soil with a shovel. The depth of digging to access the roots depends on the crop type. The collected crown root samples should be washed thoroughly with tap water before taking the image. Clean root samples can be analyzed by existing software, such as WinRHIZO and DIRT, or measured by developing new software.

### 7.2. Various Root Phenotyping Methods in Crops

Root phenotyping is a challenging task in comparison to shoot phenotyping. Nevertheless, it is necessary to aid the understanding of various aspects, such as nutrient utilization, water use efficiency, drought, and flooding stress-tolerance. Roots participate in uptake and translocation of water and nutrients. Hence, deep roots can provide resistance against drought by capturing water from deep soil layers [166,167]. Various root phenotyping methods have been established (Table 2). Essentially, root phenotypes, such as length, area, and diameter, are analyzed by collecting images [168]. Both 2D images and three-dimensional (3D) images can be used for root phenotyping, and each method has strengths and weaknesses. With magnetic resonance imaging, root morphological traits can be analyzed precisely as a 3D image, but it requires specific equipment and high expense for image collection [168]. Moreover, restricted soil conditions and plants in their early growth stages are required to collect 3D images, so, despite its precision, 3D imaging is not broadly applicable as a root phenotyping method [102,169]. For this reason, here, we will briefly describe 2D image-based phenotyping methods for field experiments. The crown root phenotyping technique is one of the promising technologies for 2D image collection under various field conditions [163]. Shovelomics has been a popular method for root crown phenotyping for the past few years because of its high throughput, with applications in QTL analysis and physiological studies [170]. Recently, root crown phenotyping has been accomplished in the context of the field environment and available resources [163].

## 8. Conclusions

Breeding plants with important root traits seem promising for developing crops for comparatively drier environments. Root traits related to drought tolerance strategies must be better understood to guide breeding programs. Similarly, with the increasing average global temperature, research efforts should be directed toward studying crop water requirements and associated physiology in hot, dry conditions [181]. It is unclear if the water uptake capacity of roots exposed to dry soil for long times and aging roots are effective for maintaining crop productivity. It has been established that the drought stress physiology of the entire plant can be used to develop an irrigation technique in partially dried roots to utilize the signaling system of the plant, which optimizes stomatal behavior, leaf growth, and shoot water status to increase the water use efficiency of the plants [182]. Research findings suggest that the presence of organic matter increases the water retention capacity of the soil, which is also nourishing for the soil microbes and, ultimately, for the plants [183]. Various research results and research questions must be communicated between plant eco-physiologists, breeders, and geneticists to improve plant productivity under drought stress. In the future, multiple root characteristics should be improved, such as water use efficiency and nutrient acquisition [184]. Practical implementation of findings from decades of research on rice roots will empower further comprehension of important traits that may impact crop productivity and profitability under abiotic stress and help cope with food insecurity.

## Figures and Tables

**Table 1:** Details of genes involved in drought tolerance.

Gene	Expression Analysis	Location of Expression	Function in Drought Tolerance	Reference
*DRO1*	Upregulated	Root apical meristem in the root tip and crown root primordia	Influences root growth angle, induces root elongation and deeper rooting	[75]
*EcNAC67*	Upregulated	Leaves and roots	Increases relative water content in leaves, delays leaf rolling symptoms, ensures better stomatal regulation during dehydration, and maintains higher root and shoot biomass	[119]
*DsM1*	Downregulated	Stamen, pistil, mature leaves and roots	Increases dehydration tolerance in the seedling stage, regulates scavenging of reactive oxygen species	[120]
*OsPYL/RCAR5*	Upregulated	Leaf blade	Stomatal closure, maintains the fresh weight of leaves	[125]
*OsDREB1F*	Upregulated	Almost all tissues, but higher in callus and panicle	Regulates the ABA-dependent signaling pathway and provides osmotic-stress tolerance	[126]
*OsDREB2B*	Upregulated	Leaf sheath, root tissues	Increases root number and length	[126]
*CYP735A*	Downregulated	Shoot	Regulates cytokinin levels	[127]
*OsNAC5*	Upregulated	Roots	Increases root diameter	[128]

**Table 2:** List of root phenotyping methods in various crops.

Crop	Trait	Method	Reference
Maize (*Zea mays*)	Root architectural traits of root crown - brace roots, number of brace roots, branching density of brace roots - number, angles, and branching density of crown root	At harvest, roots were excavated by removing a soil cylinder of 40 cm in diameter and 25-cm depth, with the plant base as the horizontal center of the soil cylinder. After root washing, clean roots were visually scored.	Trachsel et al. [170]
Arabidopsis (*Arabidopsis thaliana*)	Root system architecture - length, curvature, and stimulus-response parameters	Images were captured of Arabidopsis grown in the agarose gel condition contained in vertically-arranged plates to permit roots to grow on the surface of the medium.	French et al. [171]
Winter wheat (*Triticum aestivum*)	Root development and distribution - Number of total roots at different soil levels - Number of roots per observation depths	Field mini-rhizotrons were set up. Detailed images are available in the attached reference. Transparent rhizotubes were inserted into soil. Then, images were captured by the camera, which was located on both sides of the rhizotubes. The camera was positioned using an indexing handle at 20 observation locations in the tubes.	Cai et al. [172]
Maize (*Zea mays*)	Root morphology - axile - lateral root	Germinated seeds were transferred to moistened blotting paper in pouches. Root images were acquired by the scanner and then analyzed by WinRHIZO software.	Hund et al. [173]
Rice (*Oryza sativa*)	Root morphology - length - width - initiation angle - root tip	Rice seeds germinated in Petri plates were transplanted into glass growth cylinders containing 1.3 L of growth medium. The camera was placed in front of the growth cylinder. Image sequences were captured daily for each plant root system grown in the growth medium, consisting of 40 silhouette images taken every 9° for the entire 360° of rotation. RootReader3D software was used for the analysis of the 3D root images.	Clark et al. [174]
Sweet pea (*Lathyrus odoratus*), Sunflower (*Helianthus annuus*)	Analysis of soil aggregates to anticipate water flow toward the root	Sweet pea and sunflower seeds were planted on the surface and were grown for 30 days. An X-ray microtomography image was measured by high-resolution XMT beamline 8.3.2 at the Advanced Light Source (Lawrence Berkeley National Laboratory, USA). Transmitted X-ray light is converted to visible light using a CdWO4 single crystal scintillator, magnified by a Canon 2X lens, and imaged on a Cooke PCO 4000 CCD camera.	Aravena et al. [175]
Alfalfa (*Medicago sativa*)	Root system architecture - number of root tips - total root length - diameter - root angle orientation frequency	Alfalfa root crowns were separated from the aboveground foliage. Soil was brushed off the roots, which were then imaged in the laboratory using the RhizoVision Crown platform.	Mattupalli et al. [176]
Upland cotton (*Gossypium hirsutum* L.)	Root system architecture - total root length - average diameter of roots - number of root tips - maximum root depth - total explored area - maximum root width	Development of a root phenotyping platform, PhenoRoots, which allows for the non-invasive study of plant root system architecture. Substrate or soil-filled rhizotrons are used to grow plantlets, whose roots are directly visible through a glass plate. Pictures were taken using a digital camera and then analyzed by WinRHIZO and ImageJ software.	Martins et al. [177]
Soybean (*Glycine max* L. Merr.)	Root biomass and morphology - length - area	This study used “transparent soil” formed by the spherification of hydrogels of biopolymers. It is specifically designed to support root growth in the presence of air, water, and nutrients, and allows the time-resolved phenotyping of roots in vivo by both photography and microscopy. The roots developed by soybean plants in this medium were markedly more similar to those developed in real soil than those developed in hydroponic conditions and did not show signs of hypoxia.	Ma et al. [178]
Pea (*Pisum sativum* L.)	Root morphology - length of the tap root and lateral roots Root system architecture - number of lateral branches, branching angle representing the angle between the tap root and branched lateral roots	Measurements of root traits were performed on two phenotyping platforms. One system represented a typical high-throughput phenotyping platform for seedling root screening using agar-filled plates. The other system focused on mature root systems grown under more natural conditions (sand-filled columns) with less potential throughput. Images were analyzed using the software GrowScreen-Root	Zhao et al. [179]
Sorghum (*Sorghum bicolor* L. Moench)	Root system architecture - nodal root angle	The phenotyping platform consisted of 500 soil-filled root chambers (50 × 45 × 0.3 cm in size), made of transparent Perspex sheets that were placed in metal tubs and covered with polycarbonate sheets. Around 3 weeks after sowing, once the first flush of nodal roots was visible, roots were imaged in situ using an imaging box that included two digital cameras that were remotely controlled by two android tablets. Free software (openGelPhoto.tcl) allowed precise measurement of the nodal root angle from the digital images.	Joshi et al. [166]
Spring barley (*Hordeum vulgare* L.)	Destructive methods - total root length - root system surface - root volume - root diameter - number of tips Non‑destructive methods - root system depth - projected root surface - sum of the root lengths	The correspondence between a destructive (WinRHIZO scans) and non-destructive (RGB root imaging) method for root phenotyping using a described system was tested. The root images were analyzed after the staining of roots with powdered active charcoal. Root images were taken in the photographic room using an RGB camera. The images (JPG or TIFF files) of the plants taken in the photographic chamber were analyzed using ImageJ software. Root system scanning was performed using a specialized root scanner (STD4800 scanner) coupled with WinRHIZO Pro software (Regent Instruments, Quebec, Canada).	Slota et al. [180]
